# Surface roughness characterization using representative elementary area (REA) analysis

**DOI:** 10.1038/s41598-024-52329-4

**Published:** 2024-01-20

**Authors:** Kuldeep Singh, Nitin Paliwal, Konstantinos Kasamias

**Affiliations:** Department of Earth Sciences, Kent State University, 325 S. Lincoln St., Kent, OH 44242 USA

**Keywords:** Hydrology, Energy science and technology, Engineering

## Abstract

We proposed the Representative Elementary Area (REA) analysis method and illustrated how it is needed to evaluate representative roughness parameters of surfaces. We used mean height (Sa) roughness to study how its variations converge to a steady state as we expanded the area of investigation (AOI) using combined scan tiles obtained through Confocal Laser Scanning Microscopy. We tested quartz and glass surfaces, subjecting them to various levels of polishing with grit sizes ranging between # 60 and #1200. The scan tiles revealed a multiscale roughness texture characterized by the dominance of valleys over peaks, lacking a fractal nature. REA analysis revealed Sa variations converged to a steady state as AOI increased, highlighting the necessity of the proposed method. The steady-state Sa, denoted as ${{\text{Sa}}}_{{\text{REA}}}$, followed an inverse power law with polishing grit size, with its exponent dependent on the material hardness. The REA length representing ${{\text{Sa}}}_{{\text{REA}}}$ of glass surfaces, followed another inverse power law with polishing grit size and an indeterminate relationship for quartz surfaces. The multiscale characteristics and convergence to steady state were also evident in skewness, kurtosis, and autocorrelation length (Sal) parameters. Sal increased to a maximum value before decreasing linearly as AOI was linearly increased. The maximum Sal, termed as ${{\text{Sal}}}_{{\text{max}}}$, exhibited a linear relationship with REA. In the absence of REA analysis, the magnitude of uncertainty depended on the polishing grit size. Finely polished surfaces exhibited a 10–20% variability, which increased to up to 70% relative to the steady-state Sa with coarser polishing.

## Introduction

Surface roughness is crucial for understanding various physiochemical processes in porous media^1,2^. It significantly impacts the surface area, which controls sorption and precipitation-dissolution reactions^3–7^. Additionally, it influences colloid transport^8,9^ and chemical transport in porous media^10,11^, as well as fluid dynamics and thermal conductance^12,13^. Moreover, surface roughness affects wetting properties^14–19^ and the capillary phenomenon in porous media^20–22^. Despite its importance, the determination of mineral or sediment surface roughness has received limited attention.

When surface roughness of minerals is determined (a) it is for a quick diagnostic purpose^16–18,23,24^ and (b) its determination is limited by technique or instrument choice and method. Common methods include profilometric techniques like root-mean-square (RMS) or arithmetic mean height measurements, such as mechanical stylus, Vertical Scanning Interferometry (VSI), and Confocal Laser Scanning Microscopy (CLSM) for 2D and 3D measurements^25^. Additionally, Scanning Probe Microscopy (SPM), particularly Atomic Force Microscopy (AFM), is widely used to assess surface roughness. Further details on these techniques' advantages and disadvantages can be found in^26,27^.

Measurement techniques invariably have their limitations^25^. Traditional 2D roughness profiles cannot accommodate spatial variations^28^. High-resolution methods, such as AFM with sub-nanometer vertical resolution, sacrifice scan area, often limiting it to less than 100 µm^2^. Optical techniques, like CLSM, offer larger scan areas depending on magnification, for example, CLSM can scan 129 (µm) × 96 (µm) area at 10 nm vertical resolution using a 100 × objective lens. Additionally, combining tile scans to create a more extensive area of investigation (AOI) is a viable option, although it's underutilized in most AFM and CLSM software.

Insufficient AOI often leads to inaccurate roughness assessments, resulting in errors^25,29,30^. All roughness parameters are known to explicitly depend on the scale of measurement or scan size^12,25,26,28,29,31^ because long wavelengths exist on rough surfaces longer than the scan size. Instruments with different resolutions and scan sizes yield different values of roughness parameters^32^. Whitehouse and Archard^33^ highlighted the limitation of a single sampling interval, indicating that it depends on the correlation length. The various terms, such as AOI, scan size, sampling length, sampling interval, and sampling window are interrelated, albeit the latter two can be subsets of the total AOI or scan size.

Many advances in surface characterization have arisen from research on mechanical wear and friction of engineered surfaces^34,35^ and the analysis of roughness in joints and fractures in rock mechanics^36,37^. To study the topographic morphology of rough surfaces, various approaches can be employed, such as statistical, fractal, and directional methods^28^. Statistical characterization may include parametric and functional methods.

Parametric methods provide single values for parameters, e.g., RMS or mean height (Sa), skewness, and kurtosis over a finite surface. Functional methods involve determining the autocorrelation function (ACF), correlation length, and power spectral density (PSD), allowing roughness characterization across a wide range of wavelengths within subsets of a finite surface (Table 1). Fractal characterization is based on the premise that roughness asperities are nested within smaller asperities, creating a hierarchical structure^12,32,38^. Directional characterization aids in understanding the anisotropy of roughness in different directions, which is relevant to surfaces with preferred orientations, such as faults or joints.

**Table 1:** Notations for parameters and abbreviations used.

S.no	Symbol	Name
1	REA	Representative elementary area
2	Sa	Arithmetic mean height roughness (2D)*
3	Ra	Arithmetic mean height roughness (1D)*
4	AOI	Area of investigation
5	${{\text{Sa}}}_{{\text{REA}}}$	Steady-state Sa
6	L	Length of AOI
7	ACF	Autocorrelation function
8	Sal	Autocorrelation length
9	${{\text{Sal}}}_{{\text{max}}}$	Maximum Sal
10	Sp	Maximum peak height
11	Sv	Maximum valley depth
12	grit #	Polishing grit size number
13	Sk	Skewness
14	κ	Kurtosis
15	REA, *length*	*x*-direction length of REA
16	CLSM	Confocal laser scanning microscope

*In accordance with ISO 25178^1^ standard.

The dependence of roughness parameters on the length scale renders statistical parameters insufficient for determining representative roughness characteristics^32^ unless their scale independence is established^25^. ACF and correlation length^25,33^ indicate the scale at which statistical parameters become scale-invariant. However, correlation length has been shown to increase with sampling intervals or length scales^25,39,40^, exhibiting uncertainty in their use to determine scale-invariant roughness parameters. The Fast Fourier Transform (FFT) of ACF gives the Power Spectrum Density (PSD) enabling the evaluation of waviness and unevenness, including long and short wavelength roughness of surfaces^28^. While PSD is useful for characterizing surface topography with shorter wavelength roughness^12^, it faces challenges in detecting large wavelength characteristics^28^. In contrast, longer wavelengths significantly impact surface roughness^30^. Additionally, the reliable calculation of PSD^38^ and its application to non-stationary surfaces is challenging^28,41^, including the determination of the sampling window^26^ for non-periodic surfaces^38^.

In contrast, fractal analysis of surface roughness is desired^41^ because it offers a scale-independent characterization^32^, eliminating concerns related to scan size or resolution^38^. Fractal analysis aims to establish a power-law relationship, such as between RMS height and the sampling window or scan size^12^. This relationship is characterized by an exponent known as the Hurst exponent^28^. However, surfaces are known to demonstrate the scale-invariant property over a limited range of length scales in practice^12,41^. Gujrati et al. illustrated a power-law fractal relationship at smaller length scales or scan size, whereas Fardin et al.,^37^ showed the Hurst exponent asymptotes to a constant at larger length scales. The power-law fractal relationship tends to a ‘roll-off’ to a constant value beyond a certain larger wavelength^35^ or scan size^12^.

We postulate that the minimum length scale or wavelength at which the power-law relationship rolls off to a constant represents the threshold for determining statistical roughness parameters, such as RMS height or mean height. However, not all surfaces, particularly non-natural surfaces, exhibit fractal characteristics, leading to ambiguity in determining representative surface roughness parameters. In the absence of systematic testing for length scale independence of the roughness parameter, errors can range up to 30%^25^ or even exceed an order of magnitude^12^.

When the representative surface roughness parameter is unknown, conflicting relationships can emerge between the roughness parameter and related phenomena such as adhesion, wettability, friction, and hydro-mechanical response^12,17,28,40,42^. For example, contradictory experimental data regarding the roughness parameter and contact angle have been reported for both non-geologic material surfaces^17,42,43^ and geologic mineral surfaces^16,17^.

The objective of this study is to determine a length-scale-independent representative surface roughness parameter. To achieve this, we polish quartz and glass surfaces with six different grit sizes from coarse to fine, following a thin-section preparation routine. We scan the surfaces using CSLM at a magnification (i.e., with a 50 × objective lens) beyond which no discernable changes in height distribution are found. A tile-scan mode is used to linearly extend 2D surface scans up to 2500 µm. We analyze statistical parameters (mean height, skewness, and kurtosis), and employ functional methods (autocorrelation function and autocorrelation length) while enlarging the AOI across the extended 2D scan tiles. We apply principles of continuum mechanics to investigate how roughness parameters evolve until they converge to a steady state. The finite area at which this steady state is observed is referred to as the representative elementary area (REA).

## Results

### Single tile scan of smoother surfaces and REA

To compare surface texture variation and demonstrate the length-scale effect on surface roughness evaluation, we begin using single tile scans, i.e., field of view (FOV) of 196 µm × 254 µm) of the finest polished quartz and glass, frosted glass, and clear glass surfaces. The results obtained from these single-tile scans highlight the potential for misinterpreting roughness parameters and emphasize the necessity of conducting REA analysis on larger areas. Engineered clear and frosted glass surfaces were included in this analysis, as they were expected to exhibit no length scale dependence in roughness parameters. We compared these surfaces to the finest polished quartz and glass (grit #1200), anticipating similar length scale independence.

**Figure 1:**
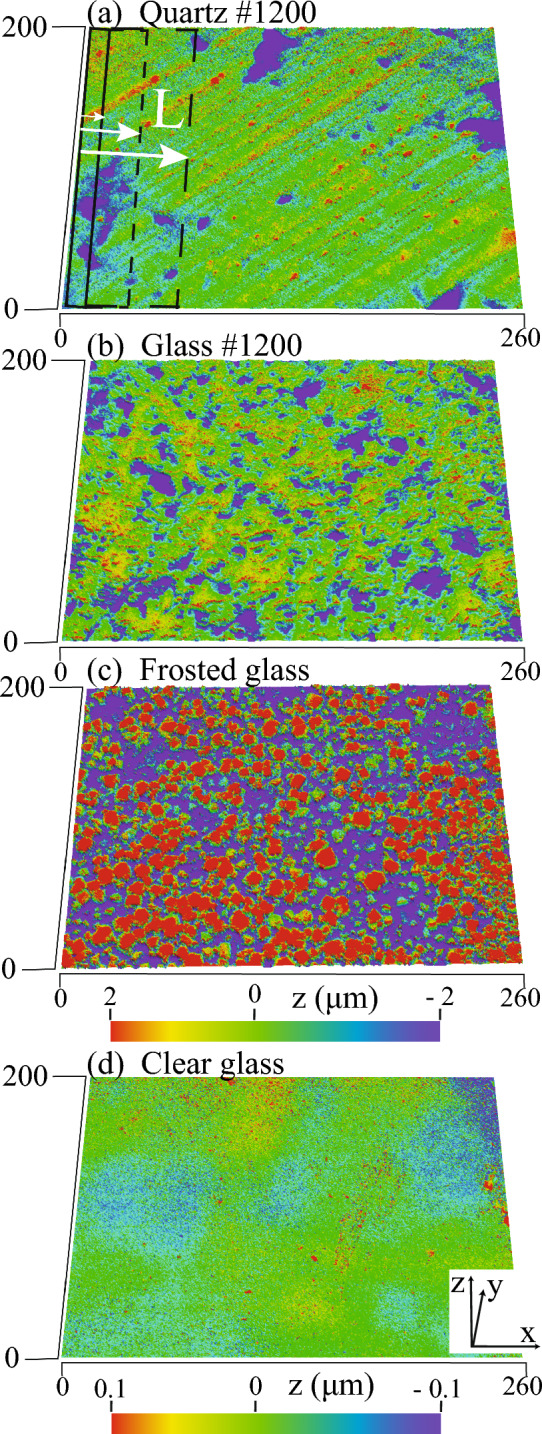
Single tile scans comparing surface texture between the finest polished (**a**) quartz and (**b**) glass surfaces and engineered (**c**) frosted glass and (**d**) clear glass surfaces.

The polished quartz and glass surfaces exhibit comparable levels of asperities but differ in spatial pattern due to the two different polishing routines discussed in the methods section. Figure 1 provides a comparison of asperities between polished quartz and glass, frosted glass surfaces using a single scale bar. A unique scale, however, is required for the clear glass surface to reveal its fine-scale asperities (Fig. 1d).

**Figure 2:**
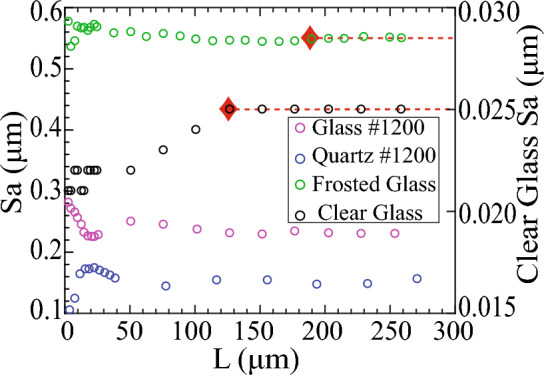
Mean height, Sa variation with sample length, L within single scan tiles shown in Fig. 1.

All single-tile surfaces exhibit randomly distributed asperities (Fig. 1), implying that a significantly smaller area than the single-tile area is sufficient to evaluate a representative roughness parameter. The mean height of these asperities, however, shows a clear length scale dependence (Fig. 2). The mean height (Sa) is the average of the absolute height values (*z*) at locations (x, y) within the specified evaluation area or AOI, calculated in accordance with ISO 25178^1^ as:

1 $${\text{Sa}}=\frac{1}{A}\iint \left|z \left(x,y\right)\right| dxdy$$

where $A$ is the sampling area, and $z \left(x,y\right)$ is the ordinate or height at a given (*x*, *y*). We assess the length scale dependence of the mean height roughness parameter (Sa) by incrementally expanding the sample window or AOI along the *x*-direction, starting at *x* = 0. The size of the sample window in the *y*-direction is kept constant so that an increase in the sample window is the same as an increase in length, L (Fig. 1a).

Engineered clear and frosted glass surfaces display variation in Sa with sample length until reaching L ~ 125 (µm), after which Sa attains a steady state. We observed no evidence of fractal or power-law scaling between Sa and L. The steady-state values are highlighted by the red dashed line in Fig. 2. The L ~ 125 (µm), marked by red diamonds (Fig. 2), represents the minimum sample length or REA required to assess the representative mean height (Sa) roughness, even for the engineered clear glass surface. Notably, this sample length, L ~ 125 (µm), significantly exceeds the capabilities of methods like AFM.

In the case of polished quartz and glass surfaces (i.e., grit #1200), Sa also exhibits variations with sample length, which converge to a steady state beyond L ~ 125 µm. However, these steady values of polished surfaces are a false account of the representative Sa since larger wavelength asperities have yet to be included in determining the REA from linearly combined scan tiles, as discussed in section "REA analysis using mean height (Sa)".

### Surface texture from linear tile scans

**Figure 3:**
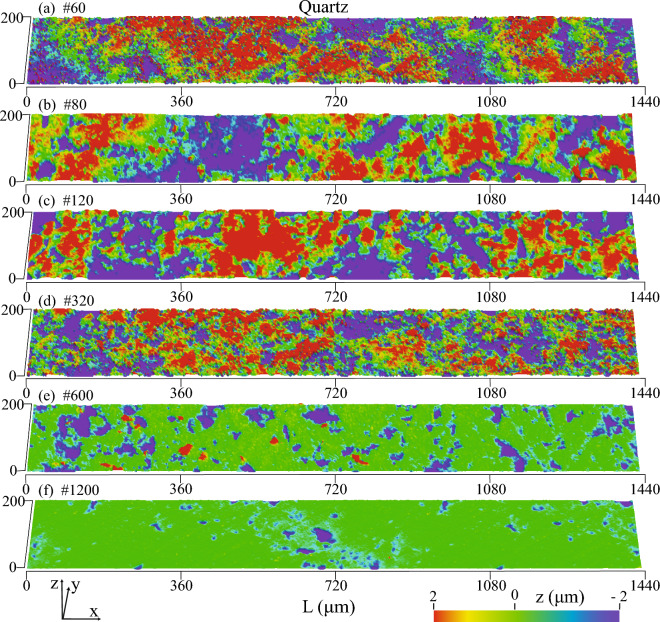
A linear combination of tile scans showing longer-wavelength surface texture variation associated with differences in the magnitude of polishing (**a**–**f**) on quartz surfaces.

**Figure 4:**
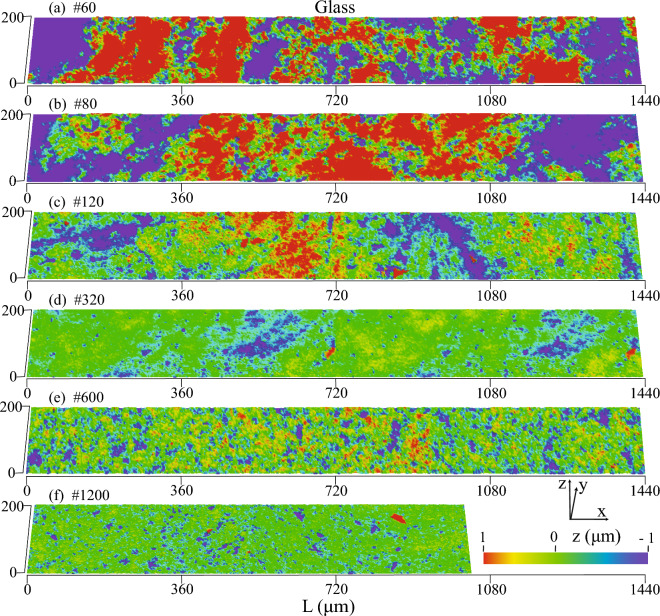
A linear combination of tile scans showing longer-wavelength surface texture variation associated with differences in the magnitude of polishing (**a**–**f**) on glass surfaces.

We utilized the tile-scan mode of CLSM to obtain large scan areas for determining representative roughness parameters of polished surfaces that remain steady as the length or AOI increases. Up to ten single tile scans were linearly combined in the *x*-direction with a 10% overlap to reach a total length of 2500 µm. Since the polishing was uniform in all directions, a linear extension of the scan area was expected to be sufficient to evaluate representative surface roughness characteristics. However, for visual clarity, Figs. 3 and 4 display six combined tiles.

The combined quartz surfaces, polished with a sequential polishing method, revealed a repeating nature of larger wavelength asperities, illustrating the multiscale nature of surface roughness (Fig. 2). The magnitude of these asperities decreased with increasing polishing fineness, from grit #60–#1200. Notably, coarse quartz polishing (grit #60) exhibited roughly equal proportions of peaks and valleys. Subsequent polishing steps reduced the magnitude of peaks and their repeating frequency; for example, sequential polishing to grit #600 almost eliminated peaks, and sequential polishing to grit #320 reduced the repeating nature of peaks (Fig. 3e,f). The characteristics of valleys, however, persisted in the surface texture evolution during polishing.

Similarly, the combined glass surfaces, polished with an individual polishing method, also revealed repeating large-wavelength asperities (Fig. 4), with the magnitude of peaks and valleys being approximately half that of quartz surfaces. Polishing glass surfaces with grit # ≤ 320 resulted in asperities with regions of peaks and valleys spanning longer lengths, while grit # ≥ 600 led to random variations in peaks and valleys, indicating the absence of multiscale roughness (Fig. 4e,f). Additionally, all individually polished glass samples showed a similar distribution of peaks and valleys, highlighting variations in surface texture development compared to quartz samples, influenced by sample crystallinity, hardness, and polishing method.

**Figure 5:**
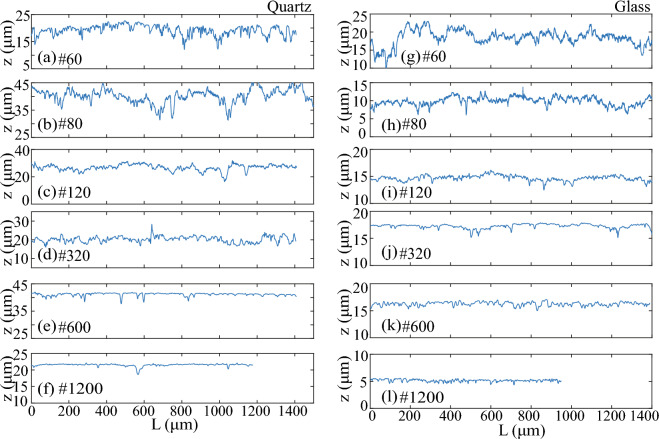
1D roughness height profiles from the middle (i.e., *y* = 98) of combined scan tiles, which are displayed in Figs. 3 and 4.

Lastly, we presented 1D height profiles from combined scan tiles for comparative analysis (Fig. 5). While recognizing the limitations of using 1D profiles for roughness parameter determination^34^, we explored whether the estimated 1D mean height (Ra) roughness parameter from significantly longer profiles aligned with Sa from 2D surfaces. We examined three 1D profile sections from the bottom, middle, and top locations of 2D surfaces. The 1D profiles of quartz samples from the middle section illustrated how sequential polishing diminished the magnitude and frequency of peaks and valleys, with differences observed up to grit #320 (Fig. 5a–f). In contrast, the 1D profiles from glass samples exhibited shorter and longer length variations (Fig. 5g–l), highlighting material-specific differences in surface texture development between the two materials. We further used mean height roughness determined from these 1D profiles to evaluate the uncertainty presented in section "Sa uncertainty in the absence of REA analysis".

### REA analysis using mean height (Sa)

**Figure 6:**
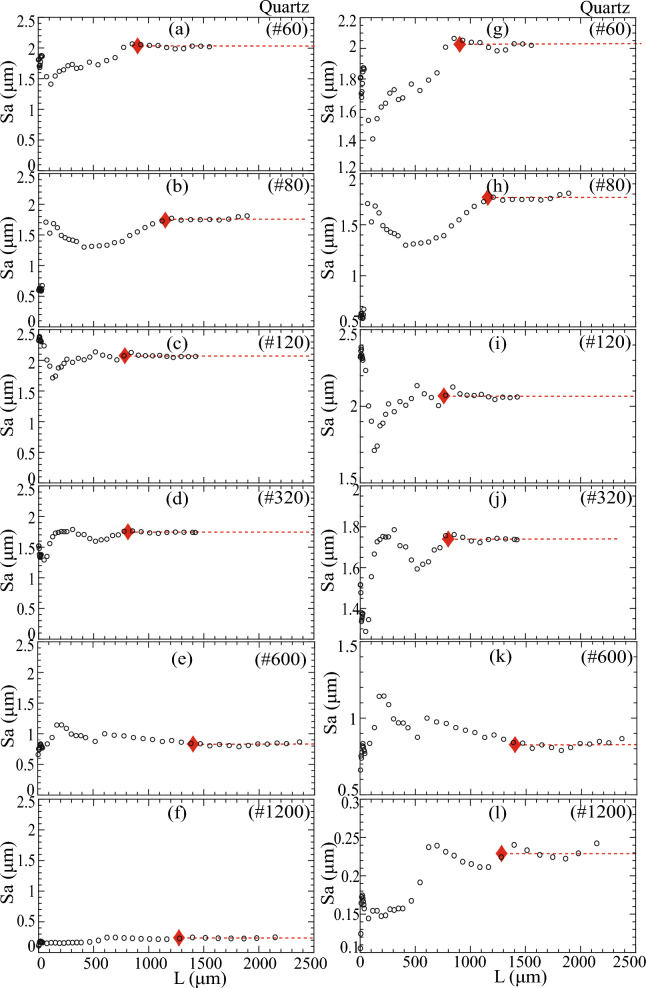
Quartz surface Sa variation with length, L. Steady state Sa is denoted by red dashed lines. REA is marked by red diamonds. Figures to the left (i.e., a to f) use a constant *y*-axis for comparison, whereas figures on the right (i.e., g to l) focus on Sa variation with variable *y*-axis limits.

**Figure 7:**
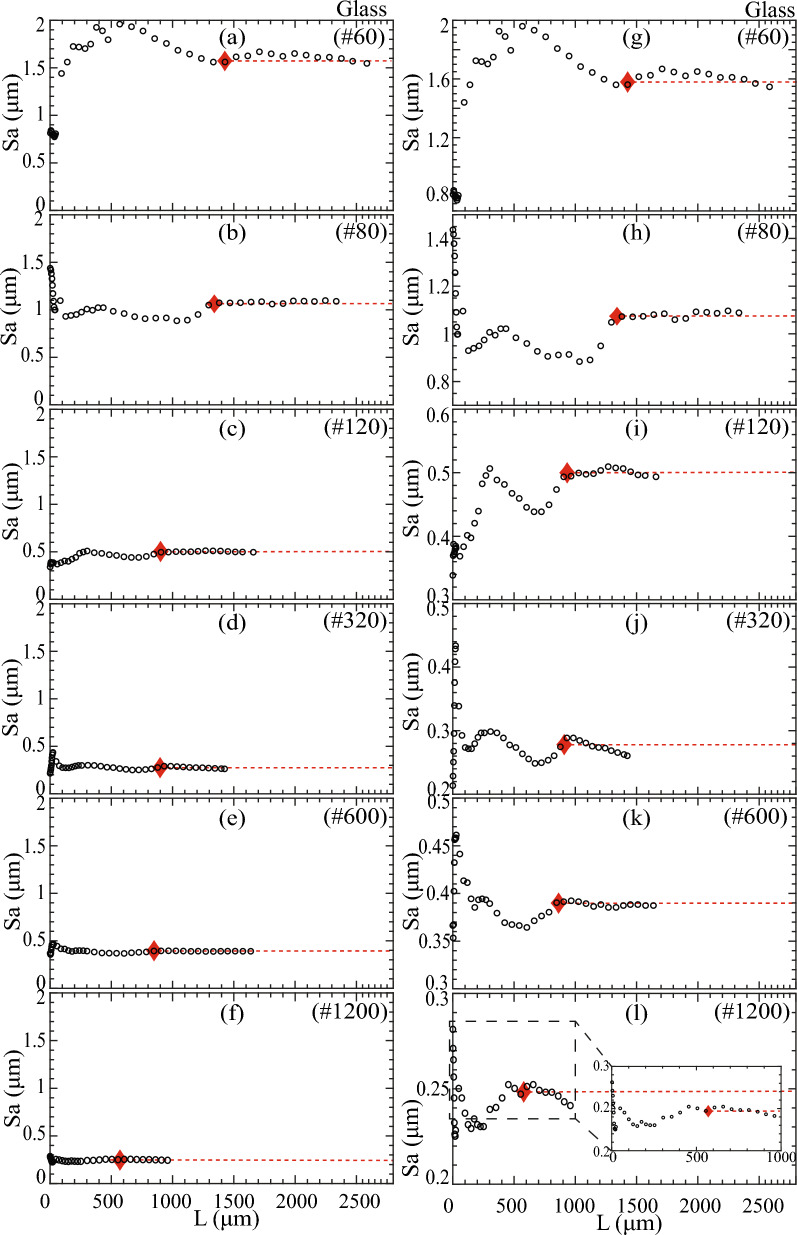
Glass surface Sa variation with length, L. Steady state Sa is denoted by red dashed lines. REA is marked by red diamonds. Figures to the left (i.e., **a**–**f**) use a constant *y*-axis for comparison, whereas figures on the right (i.e., **g**–**l**) focus on Sa variation with variable *y*-axis limits.

We proposed the REA analysis method following the concept of the Representative Elementary Volume (REV) from continuum mechanics. REA is the 2D equivalent of REV in 3D analysis. REA is defined as the area when variations in Sa asymptote to a stead state as the AOI or ‘L’ is increased, as illustrated in Fig. 1a and indicated by red diamonds in Figs. 2, 6 and 7. The determination of REA and steady-state Sa involved identifying the point beyond which the asymptotic Sa changed by less than 5% and aligned with a horizontal line. The resulting steady-state Sa was denoted as ${{\text{Sa}}}_{{\text{REA}}}$ and its corresponding area as REA. Additional information on the calculation of REA and steady-state Sa can be found in section "The REA analysis method" of the methods.

Figures 6 and 7 illustrate Sa variations with AOI or length (L) with constant and variable *y*-axis limits, allowing a comparison of Sa behavior depending on polishing intensity. The increase in AOI length, L, showed a length-dependent variation in Sa for all polished quartz and glass surfaces until L exceeded 500 µm, leading to a steady state (Figs. 6 and 7). These undulating variations in Sa indicated the absence of a fractal nature in the roughness resulting from polishing.

The magnitude of length-dependent Sa variations correlated with the polishing intensity, with coarser grit polishing (e.g., #60) exhibiting more significant Sa variations before reaching a steady state compared to finer grit polishing (e.g., #1200). Figures 6 and 7 illustrate steady-state Sa marked by red dashed lines, enabling the distinction of minor variations by considering two different *y*-axis limit modes (Figs. 6a–f and 7a–f). Similarly, Figs. 6 and 7 illustrate the AOI length at steady-state Sa or REA marked by red diamonds.

Quartz surfaces showed no specific REA length dependence on polishing grit size, while glass surfaces exhibited a decrease in REA length as the polishing grit size became finer. This difference in REA was attributed to distinct surfaces generated from different polishing methods between quartz and glass surfaces. Notably, the REA for finely polished quartz and glass #1200 surfaces was substantially larger than single tile areas, and their corresponding Sa values significantly differed. When L < REA, Sa remained substantially unrepresentative.

This REA analysis demonstrated the need for a significantly larger surface area to determine a representative roughness parameter than is typically available through methods like AFM. It clarified why discrepancies may arise when determining roughness parameters, particularly when dealing with surfaces that cannot account for larger wavelength asperities due to their limited size.

### Autocorrelation length, Sal, and REA

Whitehouse and Archard^33^ proposed matching the sampling interval to the correlation length, which accommodates long-wavelength asperities. We postulate that REA may be associated with the correlation length, offering insight into the estimation of representative roughness parameters. Here, we present an analysis of how the autocorrelation length (Sal) evolved by incrementally increasing the AOI to investigate its potential to reveal REA and roughness characteristics.

Sal is a measure of the distance over which asperities exhibit correlation with a starting point, beyond which no correlation persists. This measure is defined as the horizontal span over which the autocorrelation function (ACF) decays to nil or 0.2. The ACF quantifies the correlation of a part of the surface concerning the entire AOI. The ACF is defined as a convolution of the surface with itself, shifted by (${\tau }_{x}, {\tau }_{y}$), representing the spatial shift or ‘lag’ distance. It was computed as follows:

2 $${{\text{ACF}}(\tau }_{x}, {\tau }_{y})=\frac{\iint z \left(x,y\right) z \left(x-{\tau }_{x},y-{\tau }_{y}\right)dxdy}{\iint z {\left(x,y\right)}^{2} dxdy}$$

Sal was then calculated from ACF as:

3 $${\text{Sal}}={\text{min}}\sqrt{{\tau }_{x}^{2}+ {\tau }_{y}^{2}}$$

**Figure 8:**
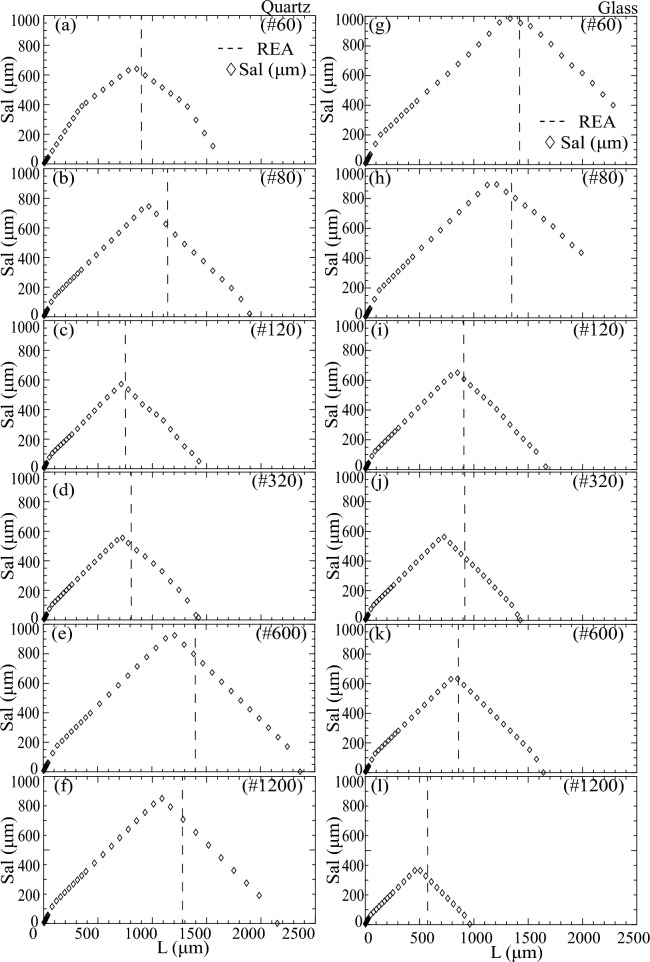
Autocorrelation length (Sal) variation with a stepwise increase in the sample window or AOI for polished quartz (**a**–**f**) and glass (**g**–**l**) surfaces. Black dashed lines denote the REA length.

For all polished quartz and glass surfaces, calculated Sal exhibited a linear increase in response to incremental AOI expansion, reaching a peak value, beyond which it decreased linearly (Fig. 8). Sal is known to characterize the wavelength structure of dominant asperity heights^39^, with smaller Sal values indicating surfaces dominated by high spatial frequency asperities, and vice versa. Thus, as the AOI expanded, Sal increased, signifying correlation over greater distances (i.e., L), until it reached a maximum correlation distance, as evident from the peaks in Fig. 8. While previous studies had reported a similar increase in Sal with sample size or AOI^25,39^, the subsequent decline in Sal beyond the peak warrants further investigation. One consideration can be that the linear increase of AOI only in the *x*-direction could create a biased shift of ${\tau }_{x}$ relative to ${\tau }_{y}$, and secondly, Sal is the minimum of all correlation distances.

**Figure 9:**
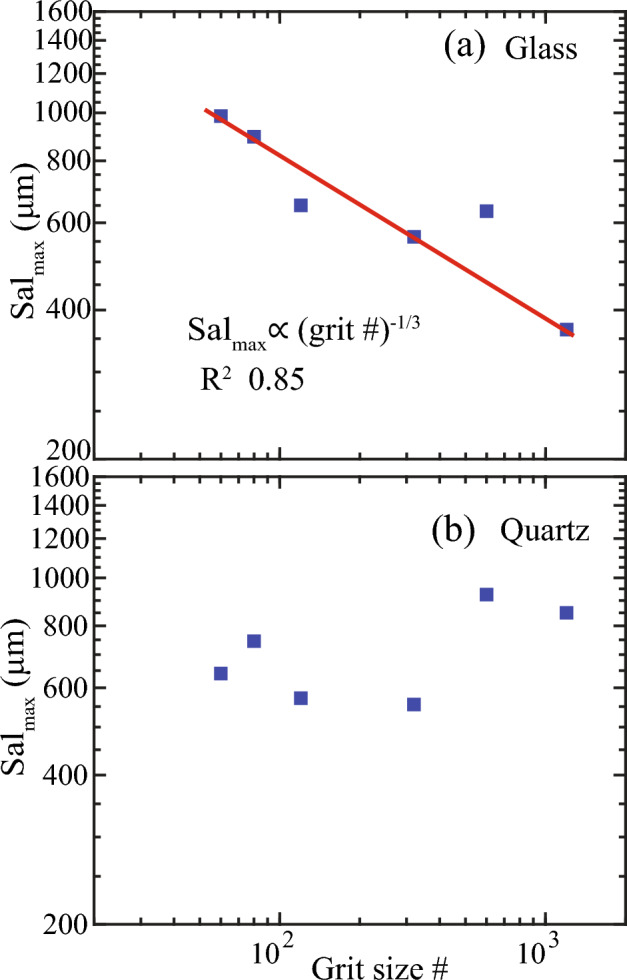
The dependence of the maximum autocorrelation length, Sal_max_ on polishing grit size #.

The peak Sal, or maximum autocorrelation length, denoted as Sal_max_, indicates the largest wavelength at which dominant asperity heights exhibit correlation or the minimum distance needed to identify all related asperities. Consequently, Sal_max_ provides a reference to REA, albeit remaining significantly smaller than REA length (L). We found that Sal_max_ followed an inverse power law with polishing grit # (Fig. 9) for glass surfaces, specifically of the form Sal_max_ $\propto 1/\mathrm{grit }{\#}^{1/3}$ (R^2^ 0.85). In contrast, no discernible trend was evident for quartz surfaces. However, it is worth noting that the REA length tended to be equal to or greater than the AOI length of Sal_max_.

### Peaks and valleys

The polishing method exerted a significant influence on roughness characteristics, particularly concerning the extent and distribution of peaks and valleys (Figs. 3 and 4). Thus, we focused on understanding the influence of polishing on areal height parameters, Sp and Sv, representing the maximum peak height and the maximum valley depth, respectively. Sp =|max(z(x,y))|, gave the height of the highest point of the surface, and Sv =|min(z(x,y))|, gave the height of the lowest point of the surface relative to the mean plane. A stepwise increment in AOI allowed for the examination of scale when the longest wavelength peaks and valleys were included in the evaluation, potentially revealing insights into REA and the impact of polishing on their relative magnitude.

**Figure 10:**
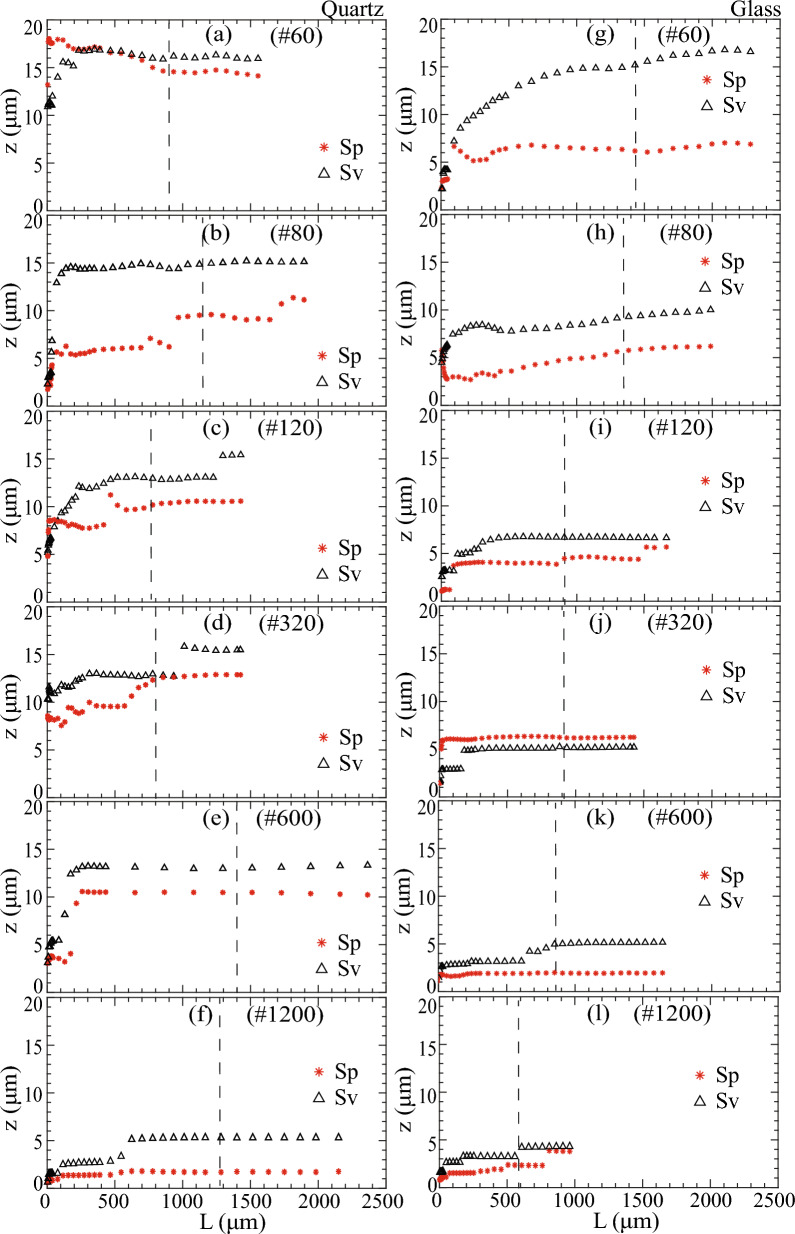
Variation in Sp and Sv for polished quartz with a stepwise increase in the sample window or AOI of quartz (**a**–**f**) and glass (**g**–**l**) surfaces. Black dashed lines denote the REA length.

Peak and valley parameters, i.e., Sp and Sv, generally increased with increasing AOI length (L), eventually reaching a steady state asymptotically (Fig. 10). Initially, several intermediate steady states corresponding to multiscale roughness features could be observed. These intermediate states ultimately converged into a final steady state as AOI length expanded. The attainment of the final steady state signified that the occurrence of the highest peak and lowest valley of all wavelengths had been comprehensively incorporated within that length or AOI.

Notably, beyond the REA length, both Sp and Sv remained constant. However, steady values for Sp and Sv could be identified at lengths less than the AOI for both quartz and glass surfaces (Fig. 10). In Fig. 10, the vertical black dashed lines denote the REA. Furthermore, the initiation of the final steady-state Sp and Sv on numerous surfaces coincided with the REA length, underscoring the importance of encompassing the highest peaks and lowest valleys of all wavelengths in the determination of REA.

Valleys surpassed peaks in size (Fig. 10). As expected, the magnitude of the peak and valley decreased as the polishing fineness or grit # increased (Fig. 10). However, the magnitude of peaks on quartz surfaces decreased significantly with increasing polishing fineness (Fig. 10a–f). As a result, polishing could be considered as removing peaks selectively (Fig. 10g–l), leaving valleys as the dominant roughness characteristics.

### Skewness and kurtosis

Skewness and kurtosis serve as height parameters that offer insights into the height distribution (*z*) of rough surfaces. Skewness, denoted as Sk, quantifies the symmetry of the height distribution within the surface topography. Positive Sk values indicate a prevalence of peaks, while negative values of Sk suggest a predominance of valleys. In instances where surface topography exhibits perfect symmetry and follows a Gaussian height distribution, Sk attains a value of zero. The calculation of Sk is expressed as follows:

4 $${\text{Sk}}=\frac{1}{{{\text{Sq}}}^{3}}\frac{1}{A}\iint \left|{z}^{3} \left(x,y\right)\right| dxdy$$

Here, Sq is the root mean square height of *z*(*x*, *y*) ordinate, calculated as;

5 $${\text{Sq}}=\sqrt{\frac{1}{A}\iint \left|{z}^{2} \left(x,y\right)\right| dxdy}$$

Kurtosis ($\upkappa$), on the other hand, serves to quantify the sharpness of the height distribution. It is a strictly positive value and indicates the extent of spikiness or bumpiness present. A high κ denotes a spiky surface, while a low κ characterizes a bumpy surface. For surfaces exhibiting a Gaussian height distribution, κ assumes a value of 3. The formula for calculating kurtosis (κ) is as follows:

6 $$\upkappa =\frac{1}{{{\text{Sq}}}^{4}}\frac{1}{A}\iint \left|{z}^{4} \left(x,y\right)\right| dxdy$$

**Figure 11:**
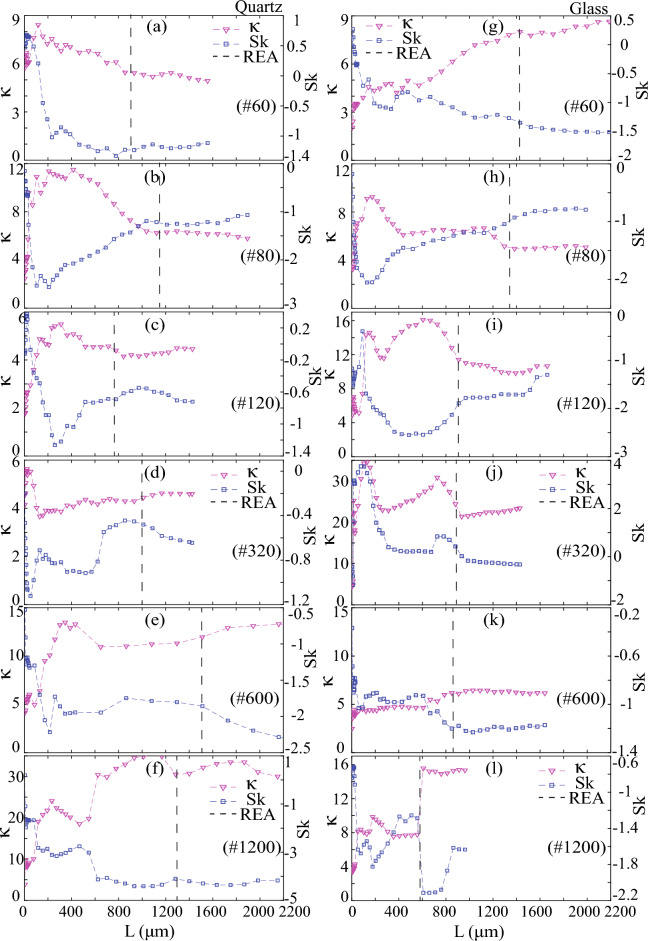
Variation in Sk and κ for polished quartz with a stepwise increase in the sample window or AOI of quartz (**a**–**f**) and glass (**g**–**l**) surfaces. Black dashed lines denote the REA length.

Skewness (Sk) and kurtosis (κ) were computed with a stepwise increase in AOI, thereby enabling an exploration of the length-scale dependency of these parameters and their potential in REA determination. The behavior of skewness (Sk) revealed a few undulations initially due to the multiscale nature of roughness on polished surfaces. It subsequently decreased as AOI increased, ultimately attaining steady negative values. This trend illustrated two significant observations: first, the length-scale dependency of Sk, and second, the prevalence of valleys as the dominant roughness feature on the studied surfaces. The magnitude of negative Sk intensified with the refinement of polishing, exemplifying how the stepwise finer polishing amplified the dominance of valleys as a roughness characteristic. Moreover, the AOI length at which Sk attained a steady state coincided with REA, thereby reinforcing REA analysis. In Fig. 11, the vertical black dashed lines denote the REA.

Conversely, kurtosis (κ), when studied with an incremental increase in AOI, exhibited a mirrored pattern in comparison to Sk. Initially displaying minor undulations, κ increased with increasing AOI, ultimately reaching steady-state positive values (Fig. 11). These steady κ values were > 3, suggesting a lognormal height distribution and the prevalence of spiky roughness, in contrast to bumpy roughness characteristics that are often associated with abrasive processes. Steady κ values grew with an increase in polishing fineness or grit size, thus indicating an augmentation in the spikiness feature for both quartz and glass surfaces. Additionally, the initiation of the final steady κ values aligned with REA, providing additional support for REA analysis alongside Sa (Fig. 11).

### REA and Sa relations with polishing grit # and Sal_max_

Our analysis of data obtained from the roughness characterization of polished quartz and glass surfaces using parametric and functional methods prompts several key questions. We aim to investigate how the REA, essential for determining the representative mean height (Sa), is influenced by the polishing grit #. Additionally, we seek to discern any connections between REA and the maximum autocorrelation length, Sal_max_. Lastly, we delve into the relationship between the representative mean height, Sa, and polishing grit #.

**Figure 12:**
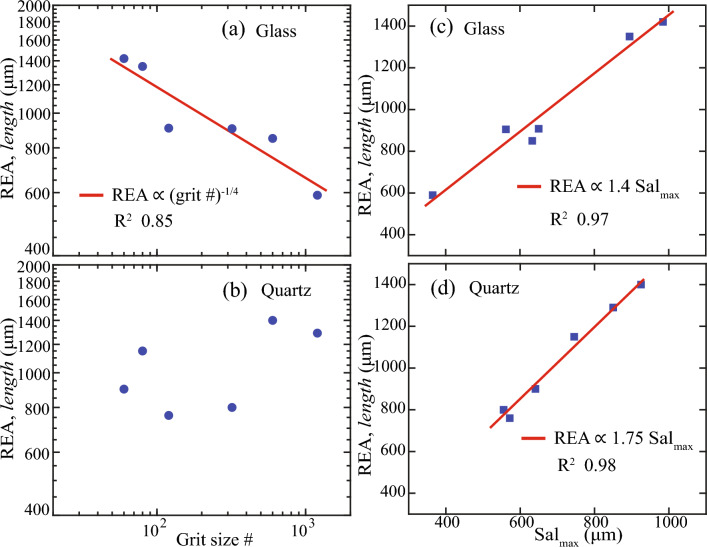
REA, *length* relationship with polishing grit size # (**a**,**b**), and maximum autocorrelation length, Sal_max_ (**c**,**d**).

In addressing these questions, we found that the smaller REA needed for finely polished glass surfaces led to an inverse power-law dependence between REA and polishing grit # (Fig. 12a), which took the form ${\text{REA}}\propto 1/\mathrm{grit }{\#}^{1/4}$ (R^2^ 0.85). However, we observed no substantial trend between REA and the polishing grit # for quartz surfaces (Fig. 12b). This divergence arose from the distinctive polishing methods employed: sequential polishing for quartz and individual polishing for glass. Consequently, the quartz surfaces exhibited an indeterminate trend due to the formation of longer-wavelength valleys when each surface was sequentially polished from coarse to fine grit #. This led to the requirement of a relatively large REA even for finely polished quartz surfaces.

As noted in Sect. 3.4, the REA length tended to be equal to or exceed the AOI length necessary to reach the maximum autocorrelation length, Sal_max_. Further examination revealed a linear relationship between L and Sal_max_ (Fig. 12c,d). The glass surfaces exhibited a shallower slope in comparison to the quartz surface. In equation form, these relationships were represented as ${\text{REA}}\propto {1.4\mathrm{ Sal}}_{{\text{max}}}$ (R^2^ 0.97) for glass surfaces and ${\text{REA}}\propto {1.75\mathrm{ Sal}}_{{\text{max}}}$ (R^2^ 0.98) for quartz surfaces. These relationships demonstrated that the REA required for calculating representative Sa surpasses the Sal_max_, as suggested in prior research^25^, and this difference varied with the wavelength of asperities present on the surface. The presence of longer-wavelength valleys on quartz surfaces necessitated a relatively larger REA than the corresponding Sal_max_, reflected by the steeper slope of 1.75. Conversely, the glass surfaces, which did not feature longer-wavelength valleys due to the ‘individual’ polishing method, required a shorter REA than quartz surfaces, albeit still longer than the corresponding Sal_max_, as indicated by the gentler slope of 1.4 (Fig. 12).

**Figure 13:**
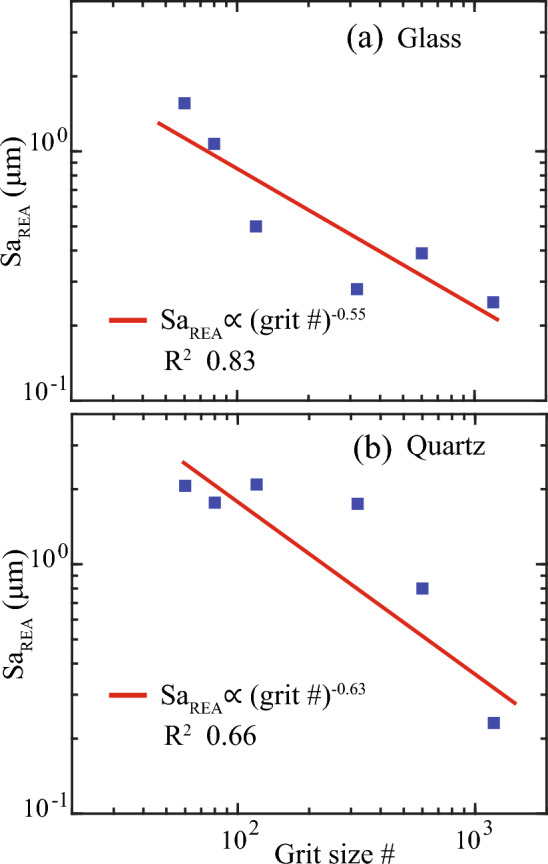
The dependence of steady-state mean height, Sa_REA_ on polishing grit size #.

It is expected that surface mean height, Sa, will depend on the degree of polishing. When considering the representative Sa, referred to as ${{\text{Sa}}}_{{\text{REA}}}$, we observed an inverse power-law relationship between ${{\text{Sa}}}_{{\text{REA}}}$ and polishing grit size # (Fig. 13). These power-law models for glass and quartz surfaces were ${{\text{Sa}}}_{{\text{REA}}}\propto \mathrm{grit }{\#}^{-0.55}$ (R^2^ 0.83) and ${{\text{Sa}}}_{{\text{REA}}}\propto \mathrm{grit }{\#}^{-0.63}$ (R^2^ 0.66), respectively (Fig. 13). This variation in ${{\text{Sa}}}_{{\text{REA}}}$ was controlled by the material’s hardness and crystallinity. Crystalline quartz has a hardness value of 7 on the Mohs scale, while amorphous glass has a hardness value of 5.5. Consequently, quartz exhibited more substantial peaks and valleys (as seen in Fig. 10), resulting in a larger Sa and a greater power-law exponent.

### Sa uncertainty in the absence of REA analysis

**Figure 14:**
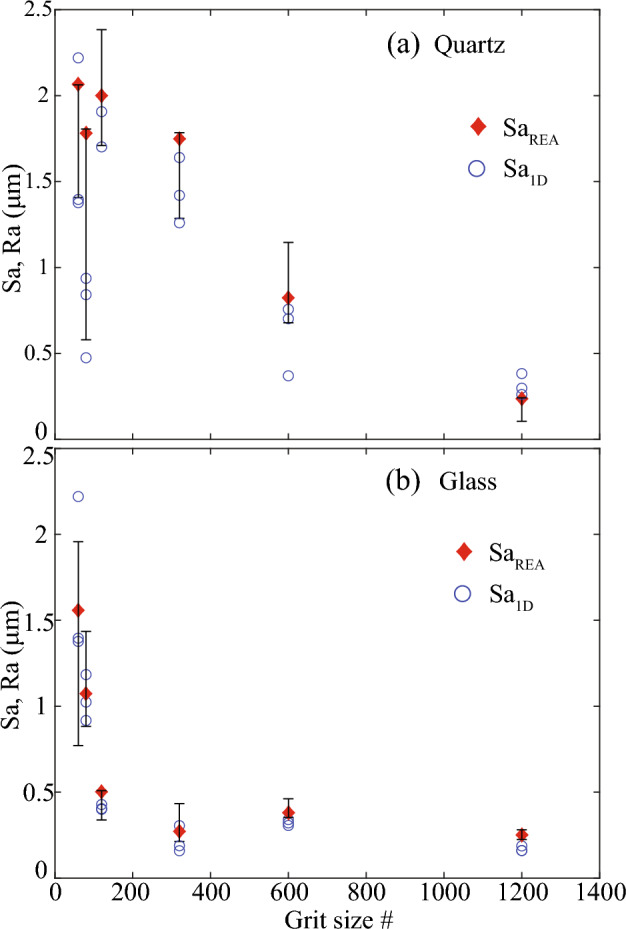
Uncertainty in surface roughness, Sa dependent on the magnitude of roughness or polishing grit size # and 1D vs. 2D analysis method.

REA analysis ensures the calculation of representative mean height, Sa. In the absence of REA analysis, the reported Sa values are susceptible to uncertainty. We were motivated to demonstrate how much uncertainty could be expected in Sa when REA analysis was not conducted. To achieve this, we computed both the maximum and minimum Sa values derived from the stepwise expansion of AOI and depicted them as error bars in Fig. 14. The red diamonds in the figure indicate the steady-state Sa or ${{\text{Sa}}}_{{\text{REA}}}$. Additionally, we introduced Sa values obtained from 1D line profiles taken at the top, middle, and bottom of 2D surfaces, allowing us to elucidate the disparity in uncertainty between 1 and 2D analyses. These Sa values from 1D profiles were represented by open blue circles, emphasizing the extent of potential variability (Fig. 14).

Our findings demonstrated that the extent of uncertainty, manifesting as variations in Sa, was magnified with coarser polishing grit sizes. Finely polished surfaces (grit # 1200) exhibited minimal Sa variability, typically deviating by 10% to 20% from ${{\text{Sa}}}_{{\text{REA}}}$. In contrast, coarsely polished surfaces yielded considerable uncertainties. For instance, quartz polished with grit # 80 registered a 2D Sa of 0.56 µm and a 1D Sa of 0.49 µm, whereas ${{\text{Sa}}}_{{\text{REA}}}$ stood at 1.77 µm. Similarly, glass surfaces polished with the coarsest grit sizes presented substantial Sa variability. Notably, quartz surfaces displayed greater Sa variability compared to glass surfaces, potentially stemming from the variation in longer-wavelength deeper valleys due to distinct polishing methods. Furthermore, the Sa values derived from 1D profiles, denoted as Sa_1D_, consistently fell below ${{\text{Sa}}}_{{\text{REA}}}$, primarily due to the underestimation of peaks and valleys inherent to the 1D profiles.

## Discussion and summary

Surface roughness determination of minerals is needed in various areas of geosciences and related engineering applications. Mineral surfaces play a pivotal role in processes such as sorption, precipitation-dissolution reactions, flow and transport phenomena, as well as multiphase saturation and transport through their influence on wettability. Despite this significance, the determination of mineral surface roughness has received inadequate attention. The most important challenge is how to determine a roughness parameter that is representative of all asperities found on a surface. Besides, extensive research demonstrates how the method, technique, or instrument used can influence roughness characterization.

Attaining high-resolution surface roughness data is crucial for examining small-wavelength asperities. However, this often results in a limited scan area of 100 µm^2^ or less. The limited scan areas cannot account for a wide range of wavelength asperities, rendering the assessment of roughness parameters contingent on the measurement scale or scan size^12,25,26,28,29,31^. When longer-wavelength asperities exist beyond the scan area, the determination of roughness becomes unrepresentative and inaccurate^12,25^.

This study aimed to establish surface roughness parameters that are representative of all asperities, which will promote reliable correlations between roughness parameters and their dependent phenomena, such as wettability^18^ or boundary slip^44^. To achieve this, we proposed the REA analysis method following the concept of continuum mechanics. Quartz and glass surfaces were polished with various grit sizes, and Confocal Laser Scanning Microscopy was employed to combine multiple single scan tiles to obtain large scan areas up to 2500 µm in length. The study focused on the mean height (Sa) parameter and its convergence to a steady-state, which defined REA.

Our study revealed that even for finely polished surfaces, single tile scans measuring 129 µm × 96 µm were insufficient for determining a representative Sa. Attempts to deduce steady-state Sa or REA from single-tile scans by incrementally increasing the AOI led to erroneous steady-state Sa values (Fig. 2). This observation emphasizes the limitations of single-tile scans in capturing the complexity of surface roughness, even in precisely polished ‘smooth’ surfaces. To provide the sample area needed to include longer-wavelength asperities and thus determine a roughness parameter that is representative of all asperities found on a surface, multiple scan tiles must be combined.

By combining surface data from up to ten scan tiles, our study unveiled a multiscale surface roughness texture influenced by polishing grit size and method. Coarser polishing introduced longer-wavelength asperities on both quartz and glass surfaces, while sequential coarse-to-fine polishing selectively removed peaks while preserving valleys on quartz surfaces. In contrast, individually polished glass surfaces exhibited a more even distribution of peaks and valleys. The stepwise finer polishing led to the selective elimination of peaks, with valleys emerging as the dominant roughness characteristics. As an example, this ratio of peaks to valleys is known to directly influence the Wenzel versus Cassie-Baxter state, controlling wettability characteristics.

Material hardness differences between glass and quartz significantly impacted surface roughness, with glass exhibiting roughly half the roughness of quartz due to its lower hardness. For example, crystalline quartz has a hardness value of 7 on the Mohs scale, while amorphous glass has a hardness value of 5.5. While the multiscale roughness texture observed might suggest a fractal nature of surface asperities, we found no evidence of a power-law relationship between the mean height (Sa) parameter and AOI length when applying the roughness-length method.

The novelty of this study lies in introducing the REA analysis method. We illustrated how REA analysis is required prior to determining representative roughness parameters. For example, this REA analysis method revealed undulating Sa variations for AOI lengths less than 500 µm, which converged to a steady state at lengths exceeding 500 µm for all surfaces. This highlights the necessity of conducting REA analysis before evaluating representative Sa. Besides, the persistence of Sa oscillations reinforces the absence of fractal roughness. Using the proposed method, we determined steady-state Sa (i.e., ${{\text{Sa}}}_{{\text{REA}}}$), which decreased with finer polishing grit, showing an inverse power-law relationship. Quartz required larger REA lengths due to persistent long-wavelength valleys induced by stepwise sequential polishing, while REA for steady-state Sa on glass decreased with finer grit, following an inverse power law. The REA analysis demonstrated that the surface area required to determine a representative roughness parameter is significantly greater than the area available, for instance when using AFM, explaining why the discrepancy in the determination of a roughness parameter could exist due to the use of length-limited surfaces that cannot take into account larger wavelength asperities^25^.

In addition to steady-state Sa, our study noted a convergence to a steady state in various parametric and functional roughness parameters, such as peaks (Sp), valleys (Sv), skewness (Sk), kurtosis (κ), and autocorrelation length (Sal). Sp and Sv displayed an asymptotic increase with AOI length, reaching a steady state at lengths analogous to REA. Sk exhibited a decline to steady-state negative values with increasing AOI length, underscoring the prevalence of valleys as prominent roughness features. The behavior of kurtosis (κ) mirrored that of Sk. A steady state κ of > 3 indicated the prevalence of spiky roughness, in contrast to bumpy roughness characteristics that are known to result from abrasive processes.

The autocorrelation length (Sal) exhibited linear increases leading to a peak value before decreasing linearly with incremental AOI length. Although prior studies have reported similar increases in Sal with sample size or AOI^25,39^, the observed reduction in Sal beyond the peak value remains a topic of further investigation. We found no correlation between Sal/L and REA, which contradicts the proposition by Nečas et al.,^25^ that a Sal/L ratio < 0.1 indicates reduced bias and sample size length for obtaining representative roughness. The maximum or peak Sal, denoted as Sal_max_, signifies the largest wavelength of correlated asperity heights or the smallest distance required to include all pertinent asperities. We found a linear relationship between Sal_max_ and REA, with a steeper slope of 1.75 for quartz and a gentler slope of 1.4 for glass surfaces. Thus, Sal_max_ clearly offered a reference to REA, although it remained consistently smaller, indicating that the REA required for determining the representative Sa can be larger than the maximum Sal^25^, depending on roughness characteristics caused by material hardness and possibly polishing method.

In the absence of the proposed REA analysis, uncertainty in reported Sa can thus be expected. We found that the magnitude of uncertainty depends on the polishing grit size. Finely polished surfaces displayed a smaller variability of 10%-20% relative to steady-state Sa, which got amplified to up to 70% of steady-state Sa with coarser polishing. Despite using significantly longer 1D profiles from combined scan tiles, the Sa from these 1D profiles remained inadequate because they underestimated the peaks and valleys, resulting in smaller Sa. Therefore, we emphasize on the significance of conducting the proposed REA analysis prior to calculating representative surface roughness parameters from 2D profiles. This proposed novel method will facilitate reliable correlations of roughness parameters with physiochemical phenomena, ultimately advancing our understanding and control of processes influenced by surface roughness in geosciences and related engineering applications.

## Methods

### Samples and preparation

The primary materials used for surface roughness evaluations were quartz and borosilicate glass samples. In addition, engineered surfaces, such as clear glass and frosted glass, were included for comparative analysis and calibration. The commonly used thin-section slides were chosen as clear and frosted glass samples, while large crystalline quartz samples were sourced from an in-house rock and mineral repository. The material properties of the glass included a density of 2.23 g/cm^3^, Poisson's ratio of 0.20, Young's modulus of 64 GPa, and a compressive strength of 915 MPa. In contrast, the quartz samples had a density of 2.65 g/cm^3^, Poisson's ratio of 0.08, Young's modulus of 95 GPa, and a compressive strength of 1100 MPa.

Besides, surface roughness can be influenced by the material’s hardness and crystallinity. Crystalline quartz has a hardness value of 7 on the Mohs scale, while amorphous glass has a hardness value of 5.5. Given these differences in crystallinity and hardness between quartz and glass, it is important to investigate how they affect the creation and evaluation of surface roughness. Prior to the polishing process, the quartz and glass samples were cut to dimensions of 1 inch × 1 inch × 0.2 inch using a wet tile saw.

### Polishing method

To achieve different magnitudes of rough surfaces, the cut-down quartz and borosilicate glass samples were polished with silicon carbide polishing discs/pads of six different grit sizes with numbers (#) of #60, #80, #120, #320, #600, and #1200.

The samples were polished using a mechanical rotatory polisher following two different polishing methods, i.e., *individual polishing* and *sequential polishing*. Glass samples were polished as desired with individual girt sizes, whereas quartz samples were polished using the thin-section slide preparation routine or sequential polishing. Sequential polishing entails polishing each sample from the coarsest grit size to the desired final grit size, followed by stepwise polishing with a finer grit size. For example, a #60 grit quartz sample was only polished with #60, whereas a #120 grit quartz sample was polished sequentially with #60, #80, and #120.

For thin-section slide preparation, sequential polishing is preferred because it removes any abrasion marks caused by the wet tile saw. Despite this well-established routine, the quartz surface will retain a memory of sequential polishing with coarser grits. Thus, we include an individual polishing method on glass surfaces to test surfaces that are not affected by other grit sizes and bring contrast to our study. Each sample with each grit size was polished for 20 min in a figure-eight polishing pattern for consistency and to achieve homogenous rough surfaces.

### Confocal laser microscopy

Surface roughness measurements were performed using a Confocal Laser Scanning Microscope, specifically the Olympus Lext OLS 3100 model. The CLSM is based on an optical method that employs a laser to illuminate a small sample volume, with a detector used to measure the light reflected or emitted from the sample. The laser beam scans across the sample in a raster pattern, and at each point, a detector measures the intensity of the light reflected or emitted from the sample.

The sample was mounted on a stage capable of digitally controlling the *x*, *y*, and *z* directions, enabling the laser beam to access different locations on a surface. Moving the objective in the *z*-direction allowed for the imaging of different sample layers, facilitating the visualization of three-dimensional structures. The objective lenses available with the Olympus Lext OLS 3100 were 5 ×, 10 ×, 20 ×, 50 ×, and 100 ×. The Lext OLS 3100 provided a maximum lateral resolution of 120 nm and a vertical resolution of 10 nm. Surface roughness was tested using all the above objective lenses, and it was concluded that the 50 × objective resolved the roughness similarly to the 100 × objective. Consequently, the 50 × objective lens was used because it offered a larger field of view (FOV) of 196 µm × 256 µm.

The Lext OLS 3100 software supported tile stitching, which allowed for the automated combination of multiple 2D scan areas using a tile scan mode. By digitally shifting the sample in the x-direction, a series of images with 10% overlap were obtained. The software then aligned and combined the scanned images to create a cohesive and continuous representation of a much larger area of the sample surface. With the tile scan mode, up to 10 tiles were combined linearly, providing a maximum surface area of 196 µm × 2500 µm for estimating roughness parameters using the REA analysis.

### The REA analysis method

Continuum mechanics theory postulates that small-scale variations in the physical properties of materials converge to a steady state or continuum as the scale is increased to a point known as the representative elementary volume (REV). Using this continuum mechanics principle, we determined the REA for mean height (Sa) that offers a representative roughness independent of the scale beyond REA (Table 1). REA is defined as the area when variations in Sa asymptote to a stead state as the AOI or ‘L’ is increased, as illustrated in Fig. 1a and indicated by red diamonds in Figs. 2, 6 and 7.

In the REA analysis, Sa was calculated by assigning a box starting from the point of origin (Fig. 1a) that outline the AOI over a subset of the total 196 µm × 2500 µm area covered by the combined scan tiles. For example, selecting an AOI of 196 µm × 5 µm from the origin, i.e., *x* = 0 (i.e., solid rectangle marked by the smallest arrow in Fig. 1a). Subsequently, the AOI was linearly increased in the *x*-direction (shown by increasing size of rectangles marked by increasing size of arrows in Fig. 1a) to reclcualte roughness parameters. This procedure was manually repeated until the AOI sampled the entire area of combined scanned tiles. To test the fractal nature of roughness, Sa was calculated by defining a small AOI initially, i.e., when *x* < 100 µm, and a slightly larger constant AOI when *x* > 100 µm.

The calculated Sa from each AOI was examined against the length of the AOI, L. Since the AOI in the y-direction remained constant, increasing AOI was equivalent to increasing L. The convergence of Sa to a steady state with an increase in AOI or L was evaluated until variations in Sa were ≤ 5%. The steady-state Sa was named Sa_REA, which provided the representative Sa. When steady-state Sa was reached, the AOI length, L, defined the REA required to calculate the representative Sa. Other parametric and functional roughness parameters, such as maximum peak height and valley depth parameters (Sp and Sv), skewness (Sk), kurtosis (κ), and autocorrelation length (Sal), were calculated similarly with a stepwise increase in AOI for roughness characterization (Table 1).

## Data Availability

The original data collected from individual scan tiles of both quartz and glass surfaces using Confocal Laser Scanning Microscopy has been archived on the Zenodo platform, a widely used open repository managed by CERN. You can access this dataset through the following link: 10.5281/zenodo.10064659. The techniques used for merging scan tiles linearly, as well as the procedures for data processing and analysis, are detailed in the methods and results sections of this manuscript and a methodology file is included on Zenodo. This data can be cited by: Singh, K., & Paliwal, N. (2023). Scan tiles obtained through Confocal Laser Scanning Microscopy for roughness characterization of surfaces. Zenodo. 10.5281/zenodo.10064659.
